# Neurological complications after pediatric cardiac surgery

**DOI:** 10.1186/s43057-021-00056-1

**Published:** 2021-09-18

**Authors:** Ergin Arslanoğlu, Kenan Abdurrahman Kara, Fatih Yiğit, Cüneyt Arkan, Ufuk Uslu, Ömer Faruk Şavluk, Abdullah Arif Yılmaz, Eylem Tunçer, Nihat Çine, Hakan Ceyran

**Affiliations:** 1 Pediatric Cardiovascular Surgery Department, Kartal Kosuyolu High Education and Training Hospital, Cevizli, 2, Denizer Caddesi, Cevizli Kavşağı, 34865 Kartal, Istanbul, Turkey; 2 Anesthesia and Reanimation Department, Kartal Kosuyolu High Education and Training Hospital, Istanbul, Turkey

**Keywords:** Cerebrovascular event, Hemiparesis, Neurological complications, Pediatric open-heart surgery, Seizure, Unconsciousness

## Abstract

**Background:**

The number of pediatric patients who survive open-heart surgery has increased in recent years and the complications seen in this patient group continue to decrease with each technological advance, including new surgical and neuroprotective techniques and the improvement in surgeons’ experience with this patient population. However, neurological complications, which are the most feared and difficult to manage, require long-term follow-up, and increase hospital costs remain a leading cause of mortality and morbidity in this cohort.

**Results:**

We evaluated the neurological physical examination, cranial computed tomography (CT), and magnetic resonance (MRI) records of 162 pediatric patients with neurological symptoms lasting more than 24 h after undergoing heart surgery in our clinic between June 2012 and May 2020. The patients’ ages ranged from 0 to 205 months, with a mean of 60.59 ± 46.44 months.

Of the 3849 pediatric cardiac surgery patients we screened, 162 had neurological complications in the early period (the first 10 days after surgery). The incidence was calculated as 4.2%; 69 patients (42.6%) experienced seizures, 17 (10.5%) experienced confusion, 39 (24.1%) had stupor, and 37 (22.8%) had hemiparesis. Of the patients who developed neurological complications, 54 (33.3%) died. Patients with neurological complications were divided into 3 groups: strokes (*n* = 90), intracranial bleeding (*n* = 37), and no radiological results (*n* = 35). Thirty-four patients (37.8%) in the stroke group died, as did 15 (40%) in the bleeding group, and 5 (14.3%) in the no radiological results group.

**Conclusions:**

Studies on neurological complications after pediatric heart surgery in the literature are currently insufficient. We think that this study will contribute to a more detailed discussion of the issue. Responses to neurological events and treatment in the pediatric group may differ compared to the adult age group. Primary prevention methods should be the main approach in combating neurological complications; their formation mechanisms should be carefully monitored and preventive treatment strategies should be developed.

## Background

More pediatric patients are surviving open-heart surgery and the neurological complications seen in this patient group are decreasing each year. Rapid advances are also being made in pediatric heart surgery: There are new surgical techniques and neuroprotective methods, and surgeons are acquiring more experience with these patients. However, neurological complications are still an important cause of mortality and morbidity today, despite all these developments [1].

The incidence of neurological complications after open-heart surgery in pediatric patients is known to differ from that of adults, as are the etiopathogenesis and risk factors of neurological complications [2]. The immaturity of pediatric patients’ central nervous system, especially in the neonatal period and infancy, increases their susceptibility to neuronal damage and stroke. Neurological complications in this group are caused by microembolization, hypoxia, hypo- and hyperperfusion, white matter injury as a result of biochemical disorders, and neuronal damage as periventricular leukomalacia [3]. Cerebral thrombus and embolism play a role in the etiopathogenesis, with associated arterial ischemic stroke being a second mechanism in the other group. Preoperative neurological problems are also seen in children with congenital heart disease, regardless of surgical complications. It should be kept in mind that abnormal brain development, chromosomal and genetic anomalies, and sequelae such as thin and coarse motor deficits secondary to previous medical interventions, attention deficit hyperactivity disorder, and speech and language disorders may develop [4].

Prospective studies on patients who underwent open-heart surgery using cardiopulmonary bypass (CPB) in the neonatal period found that their motor functions were weaker compared to the normal population even though their cognitive function was normal. This finding suggests that neurodevelopmental disorders may occur in later childhood periods in patients without cerebral damage [5].

Seizures are the most common symptom in pediatric patients who develop neurological complications after open-heart surgery. Postoperative seizures are associated with later development of severe cognitive sequelae in these patients, even though they are thought to be benign and due to postperfusion syndrome [2].

Clearly identifying the symptoms, prognostic factors, localization, and etiology of neurological complications will offer guidance in how to prevent them. Neuroprotective techniques and strategies, if applied carefully and correctly, can protect the patient from neurological sequelae that may be permanent [6]. In this retrospective study, we present the frequency, etiology, and results of neurological complications in the early postoperative period in patients undergoing pediatric cardiac surgery.

## Methods

The patients were screened retrospectively. A total of 3849 patients who underwent pediatric heart surgery between June 2012 and May 2020 in our clinic were screened. One hundred and sixty-two patients with neurological symptoms lasting more than 24 h, together with their neurological physical examination, cranial computed tomography (CT), and cranial magnetic resonance imaging (MRI) records were reviewed. Cranial imaging was performed by CT in patients who were considered to have experienced acute neurological events in our hospital, and diffusion MRI was performed in patients when deemed clinically necessary (e.g., in patients considered to have early ischemia). The patients’ CT results were reported by the radiologist, all patients were followed up by specialist neurologists, and treatment was arranged according to the recommendation of their neurologist. The study included patients with neurological symptoms in the early postoperative period. Symptoms were classified as hemiparesis, seizure, confusion, and stupor. The affected localization and sides were noted in the patients’ cranial CT records. The affected areas were recorded separately for patients affected in more than one region. The patients were divided into categories according to those who experienced a stroke, those who developed bleeding, and those whose CT results were clear or inconclusive despite having neurological symptoms.

The patients’ medical information was obtained by scanning the hospital information system and archive files. Anamnesis, genetic syndromes, history of cardiopulmonary resuscitation (CPR), history of extracorporeal membrane oxygenation (ECMO), platelet values, intensive care, ventilator, and ward times were recorded from the patients’ archive information. The preoperative neurological status of all patients was evaluated by a pediatrician and added to their files.

The operations that the patients underwent were classified according to RACHS scoring [7]. The patients’ neurological examination results, symptoms, and neurological status were recorded daily by looking at the intensive care receipts made and recorded together with the physician, pediatrician, and neurologist attending the intensive care unit. CPB non-pulsatile flow rollers were provided with a pump, and a membrane oxygenator was used in all cases. The activated clotting time (ACT) of the patients with CPB was kept above 450 s. All patients were routinely followed up with perioperative near-infrared spectroscopy (NIRS). Heparin was used for anticoagulation. The patients’ cannulations were performed selectively; the bicaval, aortic, and venous cannulation of 10 patients was performed atrially. Six patients underwent total circulatory arrest and deep hypothermia (18–20 °C).

The patients were extubated in the intensive care unit when adequate spontaneous breathing and airway reflexes were observed. Prior to any decision to extubate, each patient was evaluated for ability to perform simple orders, having an oropharyngeal temperature greater than 36.5 °C, being hemodynamically stable, and not having uncontrolled arrhythmias. In addition, arterial blood gas analyses required pH to be over 7.30, FiO2 to be less than 50%, PaO2 to be higher than 60 mm Hg, and PaCO2 to be lower than 45 mm Hg (except for single ventricle patients). The decision to transfer a patient from the postoperative intensive care unit to the ward was made following assessment of cardiopulmonary stability by an intensive care specialist, pediatric cardiac surgeon, neurologist, and pediatrician.

Number Cruncher Statistical System (NCSS) 2007 software (Kaysville, UT, USA) was used for statistical analysis. Descriptive statistical methods (mean, standard deviation, median, frequency, ratio, minimum, and maximum) were used to evaluate the study data; the distribution of the data was evaluated using the Shapiro–Wilk test. The Mann–Whitney *U* test was used to compare two groups that did not show a normal distribution of quantitative data. The χ^2^ was used to determine the relationship between qualitative data. Multiple logistic regression analysis was used to determine the effect of independent variables on the dependent variable. Significance was evaluated as *P* < 0.01 and *P* < 0.05.

## Results

A total of 3849 patients were operated on between June 2012 and May 2020 in our pediatric heart surgery clinic, and 162 patients with neurological symptoms were included in the study. The rate of neurological complications in the early period was calculated as 4.2%. The patients’ ages ranged from 0–205 months, with a mean of 60.59 ± 46.44 months. Their weights ranged from 2–80 kg, with a mean of 18.89 ± 18.08 kg. Tomography days ranged from 0–22 days, with a mean of 4.35 ± 3.22 days. Intensive care unit time ranged from 4–175 days, with a mean of 34.18 ± 31.21 days. Ward times ranged from 0–34 days, with a mean of 8.22 ± 7.14 days. CPB time ranged from 30–215 min, with a mean of 110.2 ± 54.02 min. Cross-clamp times ranged from 0–194 min, with a mean of 80.04 ± 60.72 min. Ventilator times ranged from 1–120 days, with a mean of 22.3 ± 23.89 days. Preoperative platelet values ranged from 86–663 10^3^ μL, with a mean of 306.73 ± 104.74 10^3^ μL (Table 1).

**Table 1:** Patients’ demographic details

	Mean ± SD	Min–max (median)
Age (months)	60.59 ± 86.44	0–205 (15.5)
Weight (kg)	18.89 ± 18.08	2–80 (12.1)
Days of radiological imaging	4.35 ± 3.22	0–22 (3.5)
Intensive care unit time (days)	34.18 ± 31.21	4–175 (24)
Ward time (days)	8.22 ± 7.14	0–34 (8.5)
CPB (min)	110.2 ± 54.02	30–215 (120)
Cross-clamp (min)	80.04 ± 60.72	0–194 (83.5)
Ventilator time (days)	22.3 ± 23.89	1–120 (13.5)
Preoperative platelet (10^3^ μL)	306.73 ± 104.74	86–663 (298)

*CPB* cardiopulmonary bypass

**Fig. 1:**
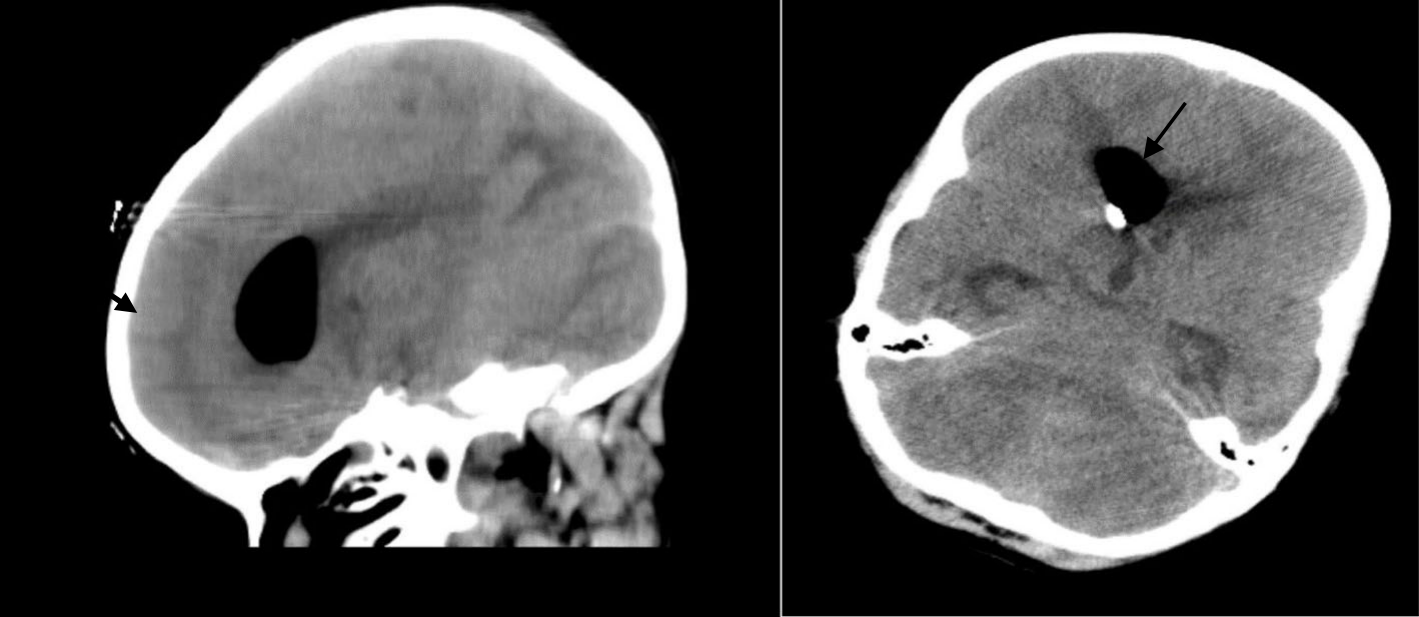
CT images of a 4-year-old patient who underwent Fontan surgery in single ventricular physiology after postoperative bleeding in the left temporal lobe (indicated by the black arrow). Extensive intracranial bleeding developed in the patient’s left temporal region on the fourth postoperative day after Fontan surgery. A drainage set was inserted by neurosurgery to relieve signs of compression

**Fig. 2:**
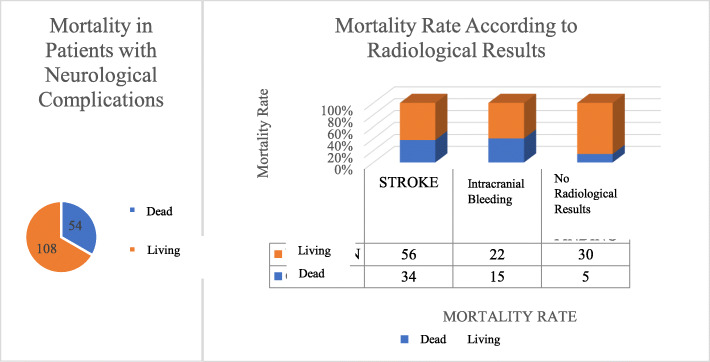
The mortality rates of patients after classification according to their radiological results

Of the patients evaluated, 37 (22.8%) had intracranial bleeding, 35 (21.6%) had no radiological results, and 90 (55.6%) developed stroke. Fifty-four patients (33.3%) who developed neurological complications died and 108 (66.7%) were discharged (Fig. 1). Thirty-four patients (37.8%) in the stroke group died, as well as 15 (40%) in the intracranial bleeding group and 5 (14.3%) in the no radiological results group (Fig. 2).

Eighty (49.4%) of the patients were female and 82 (50.6%) were male. According to the patients’ RACHS scores, 9 (5.6%) were 1 year old, 43 (26.5%) were 2 years old, 57 (35.2%) were 3 years old, 25 (15.4%) were 4 years old, 18 (11.1%) were 5 years old, and 10 (6.2%) were 6 years old.

Patients with involvement of more than one region were taken separately within two regions. Ten (4.8%) had cerebral involvement localizations in the brain stem, 56 (26.7%) had frontal involvement, 58 (27.6%) had parietal involvement, 58 (27.6%) had temporal involvement, and 28 (13.3%) had occipital involvement. Fifty-eight patients (35.8%) had multiple region involvement and 35 (21.6%) had no radiological results, while 69 patients (42.6%) had singular CT lesions. Of the CT lesions, 51 patients (31.5%) had a lesion on the left hemisphere, 38 (23.5%) had bilateral lesions, 35 (21.6%) had CT results, and 38 (23.5%) had a lesion on the right hemisphere.

Of the patients who experienced symptoms after surgery, 69 (42.6%) had seizures, 17 (10.5%) had confusion, 39 (24.1%) had stupor, and 37 (22.8%) had hemiparesis (Table 2). Regarding the interventions performed on the patients, 97 (61%) without CPR, 62 (39%) underwent CPR, 141 (87%) underwent ECMO, and 21 (13%) underwent CPR. Four patients (2.5%) had Down syndrome and 158 (97.5%) did not (see Table 2).

**Table 2:** The relationship between life status and measurements

Patient characteristics		Life status		*P*
		Dead	Living	
Sex	Female	29 (53.7%)	51 (47.2%)	0.437
	Male	25 (46.3%)	57 (52.8%)	
RACHS score	1	1 (1.9%)	8 (7.4%)	0.609
	2	13 (24.1%)	30 (27.8%)	
	3	20 (37%)	37 (34.3%)	
	4	9 (16.7%)	16 (14.8%)	
	5	6 (11.1%)	12 (11.1%)	
	6	5 (9.3%)	5 (4.6%)	
Lesion distribution	Single	19 (35.2%)	50 (46.3%)	0.001**
	Multiple	30 (55.6%)	28 (25.9%)	
	No radiological results	5 (9.3%)	30 (27.8%)	
Lesion side	Right	13 (24.1%)	25 (23.1%)	0.003**
	Left	15 (27.8%)	36 (33.3%)	
	Bilateral	21 (38.9%)	17 (15.7%)	
	No radiological results	5 (9.3%)	30 (27.8%)	
Postoperative symptom	Hemiparesis	7 (13%)	30 (27.8%)	0.001**
	Seizure	10 (18.5%)	59 (54.6%)	
	Confusion	2 (3.7%)	15 (13.9%)	
	Stupor	35 (64.8%)	4 (3.7%)	
CPR use	None	3 (5.8%)	94 (87.9%)	0.001**
	Yes	49 (94.2%)	13 (12.1%)	
ECMO use	Yes	16 (29.6%)	5 (4.6%)	0.001**
	None	38 (70.4%)	103 (95.4%)	
Additional syndrome	Down	1 (1.9%)	3 (2.8%)	0.593
	None	53 (98.1%)	105 (97.2%)	
Cerebral localization	Brainstem	6 (60%)	4 (40%)	0.079
	Frontal	17 (30.4%)	39 (69.6%)	
	Parietal	25 (43.1%)	33 (56.9%)	
	Temporal	21 (36.2%)	37 (63.8%)	
	Occipital	15 (53.6%)	13 (46.4%)	

*χ*^2^: **P < 0.01

No statistically significant differences were found regarding the patients’ age, weight, CPB, or cross-clamp values (*P* > 0.05). CT scans showed a statistically significant difference in the group without stroke/intracranial bleeding/no radiological results (*P* = 0.001; *P* < 0.01). The fact that the tomography days of the group with no radiological results were low was found to be statistically significant compared to those of the stroke and intracranial bleeding groups (*P* = 0.001; *P* < 0.01). There was a statistically significant difference in the length of time spent in the intensive care unit in the stroke/intracranial bleeding/no radiological results group (*P* = 0.001; *P* < 0.01). The intensive care unit time of the group with no radiological results was low, which was found to be statistically significant compared to those of the stroke and bleeding groups (*P* = 0.001; *P* < 0.01). Stroke/intracranial bleeding/no radiological results showed a statistically significant difference according to the group (*P* = 0.048; *P* < 0.05). The ward time of the group with no radiological results was high, which was found to be statistically significant compared to those of the stroke and bleeding groups (*P* = 0.001; *P* < 0.01). There were statistically significant differences in ventilator times for the stroke/intracranial bleeding/no radiological results group (*P* = 0.001; *P* < 0.01). The ventilator time for the group with no radiological results was low, which was found to be statistically significant compared to those of the stroke and bleeding groups (*P* = 0.001; *P* < 0.01). There were no statistically significant differences in preoperative platelet values among the patients who did not experience complications (*P* > 0.05; see Table 3).

**Table 3:** Comparison of scales according to the stroke/intracranial bleeding/no radiological results groups

		*n*	Mean ± SD	Min–max (median)	*P*
Age (months)	Stroke	90	67.51 ± 88.49	0–444 (15.5)	0.101
	Intracranial bleeding	37	40.57 ± 75.49	0–408 (12)	
	No radiological results	35	63.94 ± 90.95	1–408 (24)	
Weight (kg)	Stroke	90	20.65 ± 19.5	3–80 (12)	0.057
	Intracranial bleeding	37	13.46 ± 14.34	2–78 (11)	
	No radiological results	35	20.09 ± 17.12	3.1–61.5 (13.1)	
Days of radiological imaging	Stroke	90	4.77 ± 3.45	0–22 (4)	0.001**
	Intracranial bleeding	37	4.57 ± 3.24	1–16 (4)	
	No radiological results	35	3.03 ± 2.12	0–12 (2)	
Days in intensive care	Stroke	90	38.46 ± 34.58	5–175 (24)	0.001**
	Intracranial bleeding	37	36.05 ± 28.51	4–148 (31)	
	No radiological results	35	21.2 ± 19.89	4–84 (14)	
Ward time (days)	Stroke	90	7.68 ± 7.16	0–34 (8.5)	0.048*
	Intracranial bleeding	37	7.19 ± 7.36	0–27 (7)	
	No radiological results	35	10.71 ± 6.47	0–24 (11)	
CPB time (min)	Stroke	90	108.57 ± 51.65	30–215 (114)	0.792
	Intracranial bleeding	37	112.08 ± 66.51	30–215 (124)	
	No radiological results	35	112.4 ± 46.23	30–194 (125)	
Cross-clamp time (min)	Stroke	90	72.69 ± 59.57	0–184 (74)	0.239
	Intracranial bleeding	37	88.65 ± 69.58	0–194 (114)	
	No radiological results	35	89.83 ± 52.14	0–181 (109)	
Ventilator time (days)	Stroke	90	26.61 ± 27.28	1–120 (15.5)	0.001**
	Intracranial bleeding	37	22.62 ± 18.46	3–78 (16)	
	No radiological results	35	10.86 ± 14.49	2–68 (4)	
Preoperative platelets (10^3^ μL)	Stroke	90	313.62 ± 108.6	116–663 (299)	0.699
	Intracranial bleeding	37	290.03 ± 113.44	86–545 (296)	
	No radiological results	35	306.66 ± 83.78	176–518 (295)	

Kruskal–Wallis test: *P < 0.05; **P < 0.01

*CPB* cardiopulmonary bypass

A statistically significant relationship was found between stroke/intracranial bleeding/no radiological results and life status (*P* = 0.025; *P* < 0.05). The lowest mortality was found in the no radiological results group, while the bleeding group experienced the highest mortality rate. No statistically significant relationship was found between the stroke/intracranial bleeding/no radiological results group and sex (*P* > 0.05) or RACHS classification (*P* > 0.05). A statistically significant relationship was found between stroke/intracranial bleeding/no radiological results group in terms of finding single or multiple lesions (*P* = 0.001; *P* < 0.01), lesion side (*P* = 0.001; *P* < 0.01), and symptom (*P* = 0.001; *P* < 0.01). No statistically significant relationship was found between stroke/intracranial bleeding/no radiological results group and CPR (*P* > 0.05), ECMO (*P* > 0.05), syndrome (*P* > 0.05), or cerebral localization (*P* > 0.05).

Age value, tomography days, weight value, CPB time, and cross-clamp time were not found to show statistically significant differences according to life status after a neurological event (*P* > 0.05). The fact that the dead group had a high number of days in intensive care was found to be statistically significant compared to the living group (*P* = 0.002; *P* < 0.01). The fact that the dead group had high ventilator times compared to the living group was found to be statistically significant (*P* = 0.001; *P* < 0.01). Preoperative platelet values did not show a statistically significant difference according to life status (*P* > 0.05).

No statistically significant relationship was found between life status and sex, RACHS scoring, and localization (*P* > 0.05). There was a statistically significant relationship between life status and lesion distribution (single-multiple), lesion side, symptom, CPR, and ECMO history (*P* < 0.01).

The simple logistic regression analysis was found to be statistically significant in determining the effect of the situation without CT results on life status when the data presented in Table 4 was examined (*χ*^2^ = 8.144, *P* < 0.001). A positive and weak significant relationship was observed between the no radiological results variable and life status (*R* = 0.068, *P* < 0.001). The no radiological results independent variable in the model explained 0.06% of the total variance in life status (*P* < 0.01) and had a positive and significant effect on life status when the regression coefficients were examined (*β* = 3.769, *P* < 0.001). The simple logistic regression analysis was found to be statistically significant in determining the effect of the distribution–multiple variable on life status (*χ*^2^ = 13.532, *P* < 0.001), which had a positive and weak significant relationship (*R* = 0.080; *P* < 0.001). The distribution–multiple independent variable in the model explained 0.08% of the total variance in life status (*P* < 0.01) and had a positive and significant effect on life status when the regression coefficients were examined (*β* = 0.280, *P* < 0.001).

**Table 4:** Logistic regression analysis results for predicting life status with independent variables

	Univariate					Multivariate				
Variables	*B*	S. error	*β*	*Wald*	*P*	*B*	S. error	*Β*	*Wald*	*P*
No radiological results	1.327	0.516	3.769	6.605	0.001**					
Distribution—multiple	−1.273	0.351	0.280	13.151	0.001**					
Side—bilateral	−1.226	0.384	0.294	10.169	0.001**					
Symptoms										
Hemiparesis	0.949	0.459	2.582	4.280	0.001**	3.163	1.196	23.636	6.994	0.001**
Seizure	1.667	0.400	5.298	17.366	0.001**	3.876	1.179	48.221	10.816	0.001**
Confusion	-	-	-	-	-	4.007	1.619	54.964	6.124	0.001**
Stupor	−3.869	0.584	0.021	43.921	0.001**					
CPR	4.772	0.664	0.008	51.592	0.001**	4.893	0.842	85.26	26.088	0.001**

**P < 0.01

*P < 0.05

The simple logistic regression analysis was found to be statistically significant in determining the effect of the bilateral lesion side on life status when the data presented in Table 5 were examined (*χ*^2^ = 10.289, *P* < 0.001). A positive and weak significant relationship was observed between bilateral involvement and life status (*R* = 0.062; *P* < 0.001). The side-bilateral independent variable in the model explained 0.06% of the total variance in life status (*P* < 0.01), and the two were found to have a positive and significant effect on life status when the regression coefficients were examined (*β* = 0.294, *P* < 0.001). The simple logistic regression analysis was found to be statistically significant in determining the effect of hemiparesis on life status (*χ*^2^ = 4.819, *P* < 0.001). A positive and weak significant relationship was observed between life status and developing hemiparesis symptoms (*R* = 0.029; *P* < 0.001). The hemiparesis independent variable in the model explained 0.03% of the total variance in life status (*P* < 0.01) and had a positive and significant effect on life status when the regression coefficients were examined (*β* = 2.582, *P* < 0.001).

**Table 5:** Logistic regression analysis results for prediction of life status with independent variables

Model	Variables	Univariate					Multivariate				
		*B*	S. error	*β*	*Wald*	*P*	*B*	S. error	*Β*	*Wald*	*P*
1	No radiological results	1.327	0.516	3.769	6.605	0.001**					
	Distribution—multiple	−1.273	0.351	0.280	13.151	0.001**					
	Side—bilateral	−1.226	0.384	0.294	10.169	0.001**					
	Symptoms										
	Hemiparesis	0.949	0.459	2.582	4.280	0.001**	3.163	1.196	23.636	6.994	0.001**
	Seizure	1.667	0.400	5.298	17.366	0.001**	3.876	1.179	48.221	10.816	0.001**
	Confusion	-	-	-	-	***-***	4.007	1.619	54.964	6.124	0.001**
	Stupor	−3.869	0.584	0.021	43.921	0.001**					
	CPR	4.772	0.664	0.008	51.592	0.001**	4.893	0.842	85.26	26.088	0.001**

**P < 0.01

*P < 0.05

The simple logistic regression analysis was found to be statistically significant in determining the effect of seizure on life status in our regression analysis (*χ*^2^ = 20.469, *P* < 0.001). A positive and weak significant relationship was observed between seizure and life status (*R* = 0.119; *P* < 0.001). The seizure independent variable in the model explained 0.12% of the total variance in life status (*P* < 0.01), and had a positive and significant effect on life status when the regression coefficients were examined (*β* = 5.298, *P* < 0.001). The simple logistic regression analysis was found to be statistically significant in determining the effect of stupor on life status (*χ*^2^ = 74.562, *P* < 0.001), and a positive and weak significant relationship was observed between them (*R* = 0.369; *P* < 0.001). The insomnia independent variable in the model explained 0.37% of the total variance in life status (*P* < 0.01). The stupor variable had a positive and significant effect on life status when the regression coefficients were examined (*β* = 0.021, *P* < 0.001).

The simple logistic regression analysis was found to be statistically significant in determining the effect of CPR on life status (*χ*^2^ = 110.556, *P* < 0.001), and a positive and moderate significant relationship was observed between the two (*R* = 0.501; *P* < 0.001). The CPR independent variable in the model explained 50% of the total variance in life status (*P* < 0.01) and had a positive and significant effect on life status when the regression coefficients were examined (*β* = 85.26, *P* < 0.001).

The multiple logistic regression analysis was found to be statistically significant in determining the effect of the independent variables on life status (*χ*^2^ = 141.399, *P* < 0.001). A positive and moderate significant relationship was observed between independent variables and life status (*R* = 0.589, *P* < 0.001), and the independent variables in the model explained 58.9% of the total variance in life status.

Hemiparesis (*β* = 23.636, *P* < 0.001), seizure (*β* = 48.221, *P* < 0.001), confusion (*β* = 54.964, *P* < 0.001), and CRP (*β* = 85.26, *P* < 0.001) had positive and statistically significant effects on mortality when the regression coefficients were examined.

## Discussion

Neurological complications after pediatric heart surgery are still an important cause of mortality and morbidity today. The treatment of neurological complications is usually palliative; the main goal is to prevent them using neuroprotective methods, as there is no sequelae-free treatment for the majority of patients [8]. In our study, we investigated the prognosis of patients who developed neurological complications in the early postoperative period and the factors affecting their prognoses.

The risk of stroke is low, as atherosclerosis is less common in the pediatric population than in the corresponding adult population. The risk of stroke in the adult group is reported to be 6% in the literature [9], while among the pediatric population it is 0.54% according to Domi et al. [1]. The risk of developing neurological events was found to be 4.2%; in our study, 55.6% of the patients we evaluated had a stroke, although the rate of stroke development among all patients who underwent surgery was 2.3%.

Although there are rarely clinical signs of cranial diffuse radiological involvement in the pediatric population, the duration of neurological morbidity in these patients is much longer than that of adults [10, 11]. We included symptomatic patients in our study: patients who did not develop neurological symptoms but had imaging results constitute a limitation of our study.

Jafri et al. reported that 35 (1.75%) of the 2000 pediatric cardiac surgery patients they evaluated developed acute neurological complications, and 28 of those 35 patients (80%) presented clinical signs of seizure [4]. Of the 3849 patients who underwent surgery, 162 had neurological complications in the early period, comprising a rate of 4.2% in our patient group; 42.6% of these 162 patients (*n* = 69) had seizures, 10.5% (*n* = 17) had confusion, 24.1% (*n* = 39) had stupor symptoms, and 22.8% (*n* = 37) demonstrated symptoms of hemiparesis. One of the reasons for the high number of stroke patients may be that the mean age of our patients was older.

Trittenwein et al. argued that the effect of CPB time on neurological damage in the early postoperative period was statistically insignificant and that being above 100 mg/dL at lactate levels with postoperative acidosis was the cause of 30% of cerebral damage symptoms [12]. We found in our study that age, weight, CPB, and cross-clamp time had no statistically significant effect on mortality in patients who experienced a neurological event. Of the patients who died, 64.8% had stupor, 3.7% had confusion, 18.5% had seizures, and 13% had hemiparesis. We found that presenting with neurological complaints soon after surgery was a statistically significant predictor of mortality.

Implanting cardiac shunts (ASD, VSD) in congenital heart patients may cause paradoxical embolisms, and it is known that high hematocrit values create a predisposition to coagulopathy in the majority of these patients. Antiaggregant and anticoagulant drugs used in the postoperative period increase the risk of bleeding. However, ischemic strokes were reported to be more common than intracranial bleeding in this cohort, despite the use of antiaggregant and anticoagulant medications [13]. The rate of ischemic stroke was higher in patients who underwent CT imaging compared to the others in our study. This may be due to increased myeloid and thrombocyte blood cell series secondary to coagulopathies and hypoxia secondary to genetic syndromes, which are common in these patient groups.

Werho et al. reported that patients undergoing pediatric cardiac surgery connected to ECMO had the highest risk of stroke and other neurological complications [14]. Twenty-one of the patients we evaluated developed neurological complications during ECMO, and mortality was calculated as 29.6% in the ECMO group and 4.6% in the non-ECMO group in our study. Of these, 13 (62%) did not have ischemia, 7 (33.3%) did not have bleeding, and 1 (4.7%) did not have radiological imaging results. We think that blood-shaped element destruction and bleeding may occur following the administration of anticoagulant drugs, while ischemic events are the result of inflammatory reactions and associated increased coagulation caused by lines and solutions used in ECMO circulation.

In their study on strokes after pediatric cardiac intervention, Henzi et al. reported that patients who developed neurological complications showed symptoms after an average of 4 days and were diagnosed after an average of 2 days; they stated that hypotension, prosthetic heart material, right-to-left shunt, arrhythmias, low heart rate, and infections are risk factors [15]. In our study, the diagnosis time was 4.7 days in the stroke group, 4.5 days in the bleeding group, and 3 days in the group with no CT results, which is similar to the results reported by Henzi et al. [15].

Madhok et al. argued that the increased need for vasoactive inotropes increased pro-inflammatory cytokines with decreased heart rate and increased morbidity by reducing cerebral perfusion pressure and oxygenation [16]. We did not find a statistically significant difference between the etiology (ischemia, bleeding, etc.) and mortality of patients who developed neurological complications when we classified the patients according to their RACHS scores in our study.

It is necessary to monitor the effects of coronary bypass on the central nervous system in order to prevent cerebral damage associated with cardiac surgery. NIRS, a noninvasive method in CPB management, especially during TSA and regional low-flow perfusion, is useful in preventing neurological complications after pediatric heart surgery [17]. We routinely use NIRS monitoring in our own surgical practice.

Agha et al. found that CT and MRI results and myocardial contraction power were correlated with each other, but they did not find a statistically significant correlation with the length of intensive care stay [6]. We found a statistically significant difference between the duration of intensive care when we separated the patients into ischemic, bleeding, and no radiological results categories, in both the dead and the living groups in our study. The longest intensive care period was 38.46 days in the ischemia group, followed by bleeding (36.05 days) and no radiological results (21.2 days).

Requiring ECMO and CPR in the postoperative period increases the risk of neurological complications, and 13% of neurological complications occur after ECMO intervention after CPR, a procedure that combines the two interventions, according to Extracorporeal Life Support Organization records [18]. Mortality was found to be statistically significantly higher in the CPR and ECMO groups of patients with neurological complications in our study. Ischemic strokes are more common in both groups than in the general population, although there was no statistically significant relationship when the CT results of the ECMO and CPR groups were examined.

Andropoulos et al. investigated the factors affecting brain damage after neonatal heart surgery and presented their own treatment and prevention strategies [19]. They argued that brain damage revealed in postoperative MRI examinations of this patient group may be prenatal, postnatal, or preoperative, and emphasized the importance of detecting, diagnosing, preventing, and early treatment of brain damage in the neonatal period to optimize neurodevelopmental outcomes [19]. In our study, we found that age and patient weight had no statistically significant effect on mortality in patients who developed neurological damage. The mean age and weight of the bleeding group were found to be lower, which is thought to be due to the immature coagulation system causing a tendency to bleed in the neonatal period, although no statistically significant results were found when the ages and weights of the bleeding and stroke groups were compared.

The rate of acute neurological complications was reported to be 2–3% in the postoperative period. However, it varied greatly depending on age and modality in the postoperative period, and neurological complications have been detected in up to 50% even if postoperative imaging methods (MRI, EEG, CT) used on non-symptomatic patients revealed no clinical signs. Our knowledge of the clinical significance of these signs in asymptomatic patients is neither clear nor sufficient [20–24]. It is currently not possible to detect subclinical cases in such patients, even though the incidence of postoperative neurological complications was 4.2% in our study Gunn et al. found that the motor, language, and cognitive scores of patients who developed neurological complications after pediatric heart surgery were lower than the rest of the population in post-discharge follow-ups [2]. In our study, we found mortality rate to be 33.3%; this result can be interpreted as indicating that the remaining 66.7% have a high morbidity rate in the future, according to Gunn et al. [2].

In our study, the need for prolonged ventilation and prolonged intensive care were found to be statistically significant predictors of mortality in patients with neurological complications. The highest mortality rate (60%) was found to affect patients with brainstem involvement, even though there was no statistically significant difference between the localization of neurological sequelae and mortality in our study.

## Conclusions

Current treatment modalities, and mortality and morbidity are very high in patients with neurological complications. The number of studies on neurological complications after pediatric heart surgery in the literature remains insufficient. We think that our study will contribute to a more detailed discussion of the issue. As responses to neurological events in and treatment for this age group may differ from those of adult patients, primary prevention methods should be the main approach to combating neurological complications; these patients’ formation mechanisms should be carefully monitored, and more preventive treatment strategies must be developed.

## Limitations

This is a single-center, retrospective study. Postoperative routine cranial imaging is not performed in our hospital. Cranial CT scans were performed for all patients with symptoms upon admittance. Diffusion MRI, which provided better images of ischemic status in the early period, was applied to all patients upon recommendation by the neurology specialist. Failure to perform imaging on patients without neurological symptoms constitutes the primary limitation of this study.

## Data Availability

Data sharing is not applicable to this article as no new data were created or analyzed in this study.

## Abbreviations

ACT: Activated clotting time
CPB: Cardiopulmonary bypass
CPR: Cardiopulmonary resuscitation
CT: Computed tomography
ECMO: Extracorporeal membrane oxygenation
NIRS: Near-infrared spectroscopy
